# Dietary Calcium Intake and the Risk of Metabolic Syndrome: A Systematic Review and Meta-Analysis

**DOI:** 10.1038/s41598-019-55507-x

**Published:** 2019-12-13

**Authors:** Dan Han, Xuexian Fang, Danting Su, Lichun Huang, Mengjie He, Dong Zhao, Yan Zou, Ronghua Zhang

**Affiliations:** 1 Department of Nutrition and Food Safety, Zhejiang Provincial Center for Disease Control and Prevention, Hangzhou, China; 2 Department of Nutrition, School of Public Health, Zhejiang University School of Medicine, Hangzhou, China

**Keywords:** Metabolic syndrome, Epidemiology

## Abstract

Growing evidence has suggested a possible relationship between dietary calcium intake and metabolic syndrome (MetS) risk. However, the findings of these observational studies are inconclusive, and the dose-response association between calcium intake and risk of MetS remains to be determined. Here, we identified relevant studies by searching PubMed and Web of Science databases up to December 2018, and selected observational studies reporting relative risk (RR) with 95% confidence interval (CI) for MetS based on calcium intake and estimated the summary RRs using random-effects models. Eight cross-sectional and two prospective cohort studies totaling 63,017 participants with 14,906 MetS cases were identified. A significantly reduced risk of MetS was associated with the highest levels of dietary calcium intake (RR: 0.89; 95% CI: 0.80–0.99; *I*^2^ = 75.3%), with stronger association and less heterogeneity among women (RR: 0.74, 95% CI: 0.66–0.83; *I*^2^ = 0.0%) than among men (RR: 1.06, 95% CI: 0.82–1.37; *I*^2^ = 72.6%). Our dose-response analysis revealed that for each 300 mg/day increase in calcium intake, the risk of MetS decreased by 7% (RR: 0.93; 95% CI: 0.87–0.99; *I*^2^ = 77.7%). In conclusion, our findings suggest that dietary calcium intake may be inversely associated with the risk of MetS. These findings may have important public health implications with respect to preventing the disease. Further studies, in particular longitudinal cohort studies and randomized clinical trials, will be necessary to determine whether calcium supplementation is effective to prevent MetS.

## Introduction

Metabolic syndrome (MetS) is characterized by a constellation of interacting risk factors including impaired glucose tolerance (IGT), atherogenic dyslipidemia, elevated blood pressure, and visceral adiposity. These factors contribute to type 2 diabetes mellitus(T2D), cardiovascular disease (CVD), and other interrelated diseases^1,2^. The global incidence of MetS is continuing to rise over the past decades. The age-adjusted prevalence of MetS in the United States increased from 29.2% to 34.2% from 1999 to 2006^3^. Asian populations also showed the similar trend^4^. Given its strong link to several chronic diseases, preventing MetS has been an important public health problem.

Calcium, an essential element, is the most abundant ion in human body. It is mainly stored in bones in the form of hydroxyapatite^5^. Serving as a ubiquitous signaling molecule with diverse role, calcium is also involved in a wide variety of physiological processes outside of the skeleton, including neuronal transmission, muscle contraction, organelle communication, hormone secretion, fertilization, and cell growth^6^. Previous evidence from meta-analysis indicated that calcium may reduce the risk of CVD and some types of cancer^7,8^. In the recent dozen years, a growing body of epidemiological studies evaluated the association between dietary calcium intake and the risk of MetS. However, the results of these studies were not consistent, and the overall conclusion still remains controversial.

To our knowledge, there is no meta-analysis has been performed before to study the putative association between dietary calcium intake and the risk of MetS. Hence, we conducted the current systematic review and meta-analysis in order to quantify the dose-response relationship between dietary calcium intake and MetS risk.

## Results

### Study selection

**Figure 1:**
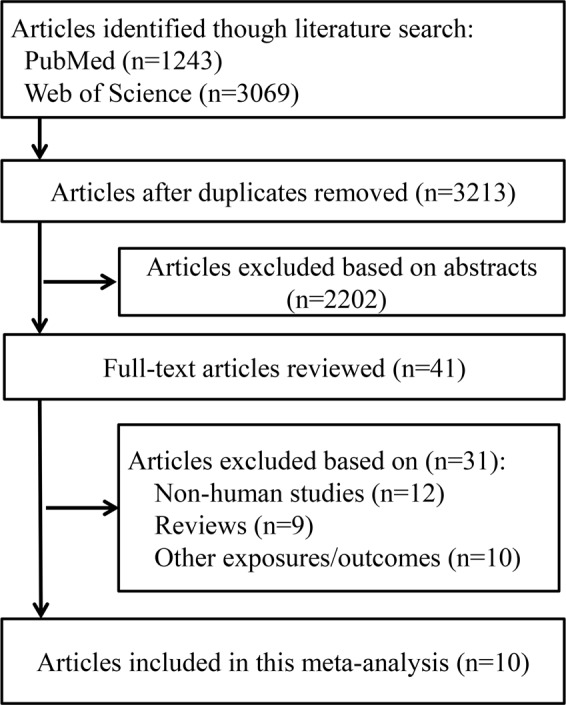
Flow diagram of literature search and study selection.

Figure 1 reports the bibliographic research process. In total, we identified 3,609 articles from Web of Science, and 1,243 articles from PubMed using our search strategy. Duplicate studies and studies that did not fulfill the inclusion criteria were excluded. Finally, we included 10 articles in our meta-analysis: 8 cross-sectional studies^9–16^ and 2 prospective cohort studies^17,18^.

Table 1 shows the detailed characteristics of the 10 studies included in this meta-analysis. In total, these studies, which were published from 2005 through 2018, included 63,017 participants and 14,906 MetS cases aged 20 years and older. Five of the studies were performed in Korea^10–12,15,16^, two studies were conducted in United States^13,18^, and the others were performed in Saudi Arab^9^, France^17^, and Australia^14^, respectively. The definition of metabolic syndrome in all these studies either used the International Diabetes Federation (IDF) criteria or the National Cholesterol Education Program (NCEP) ATP III criteria. Quality assessment for the studies yielded an average score of 7.7 (0.9) (Tables 2–3), and the quality of all the 10 studies was deemed moderate or high (score of 6 or above).

**Table 1:** Characteristics of the included studies (n = 10) regarding the association between dietary calcium intake and MetS.

Author, Year	Study Name	Design	Location	Cases/Participants	Sex	Age (y)	Follow-up (y)	Assessment	Adjustment
Al-Daghri *et al*., 2013	N/A	CS	Saudi	72 (IDF)/185	M/F	19–60	/	24-h recall	Age, BMI, physical activity
Cho *et al*., 2009	KNHANES 2001–2005	CS	Korea	2479 (NCEP)/9341	M/F	>20	/	24-h recall	Age, BMI, marital status, education, alcohol, smoking, exercise, energy intake
Fumeron *et al*., 2011	DESIR	PC	France	667 (IDF) or 452 (NCEP)/3435	M/F	30–65	9	FFQ	Sex, age, BMI, smoking, total fat intake, physical activity
Kim *et al*., 2017	KNHANES 2008–2011	CS	Korea	2762 (NCEP)/14705	M/F	>20	/	24-h recall	Total calorie intake, calcium supplement intake, age, living area, education, income, occupation, marital status, alcohol, smoking, exercise level, stature, BMI
Lim *et al*., 2017	N/A	CS	Korea	49 (NCEP)/143	M/F	58.0 ± 9.3	/	3-d recall	Age, sex, BMI, alcohol, smoking, exercise
Liu *et al*., 2005	WHS	PC	US	1039 (NCEP)/10066	F	≥45	8.8	FFQ	Age, smoking, exercise, total calories, alcohol, multivitamin use, parental history of myocardial infarction
Moore-Schiltz *et al*., 2015	NHANES	CS	US	3579 (NCEP)/9148	M/F	>20	/	24-h recall	Sex, age, ethnicity, education, income, total energy intake and fiber intake.
Pannu *et al*., 2016	VHM	CS	Australia	735 (IDF)/3403	M/F	15–75	/	24-h recall	Age, sex, country of birth, income, education, smoking, energy intake, physical activity, body weight, alcohol, dietary fiber
Shin *et al*., 2015	MRCohort	CS	Korea	2018 (NCEP)/6375	M/F	>40	/	FFQ	Age, education, marital status, exercise, glycemic load, intake of fat, fiber, sodium and energy
Shin *et al*., 2016	KNHANES 2010–2012	CS	Korea	1506 (NCEP)/5946	M	>20	/	24-h recall	Age, BMI, smoking, alcohol, income, education, residential area, physical activity, energy intake, eGFR, serum 25(OH)D level

Abbreviations: BMI, Body mass index; CS, Cross-sectional; DESIR, Data from the Epidemiological Study on the Insulin Resistance; FFQ, Food frequency questionnaire; IDF, International Diabetes Federation; KNHANES, Korea National Health and Nutrition Survey; MRCohort, Multi-Rural Communities Cohort Study in Rural Communities; N/A, Not Available; NCEP National Cholesterol Education Program; PC, Perspective cohort; US, United States; VHM, Victorian Health Monitor; WHS, Women’s Health Study.

**Table 2:** Quality assessment of included perspective cohort studies.

Author, year	Selection				Comparability	Outcome			Overall quality
	Representative of exposed cohort	Selection of controls	Exposure ascertainment	No history of disease	Comparable on confounders	Outcome assessment	Adequate follow-up time (>5 years)	Follow-up rate (>80%)	
Fumeron *et al*., 2011	0	1	1	1	2	1	1	0	7
Liu *et al*., 2005	0	1	1	1	2	1	1	0	7

**Table 3:** Quality assessment of included cross-sectional studies.

Author, year	Selection				Comparability	Outcome		Overall quality
	Representative of the sample	Sample size	Non-respondents	Exposure ascertainment	Comparable on confounders	Outcome assessment	Statistical test	
Al-Daghri *et al*., 2013	0	0	0	2	2	2	1	7
Cho *et al*., 2009	1	0	0	2	2	2	1	8
Kim *et al*., 2017	1	1	0	2	2	2	1	9
Lim *et al*., 2017	0	0	0	2	1	2	1	6
Moore-Schiltz *et al*., 2015	1	0	0	2	2	2	1	8
Pannu *et al*., 2016	1	1	0	1	2	2	1	8
Shin *et al*., 2015	1	1	0	2	2	2	1	9
Shin *et al*., 2016	1	1	0	1	2	2	1	8

### Dietary calcium intake and MetS

**Figure 2:**
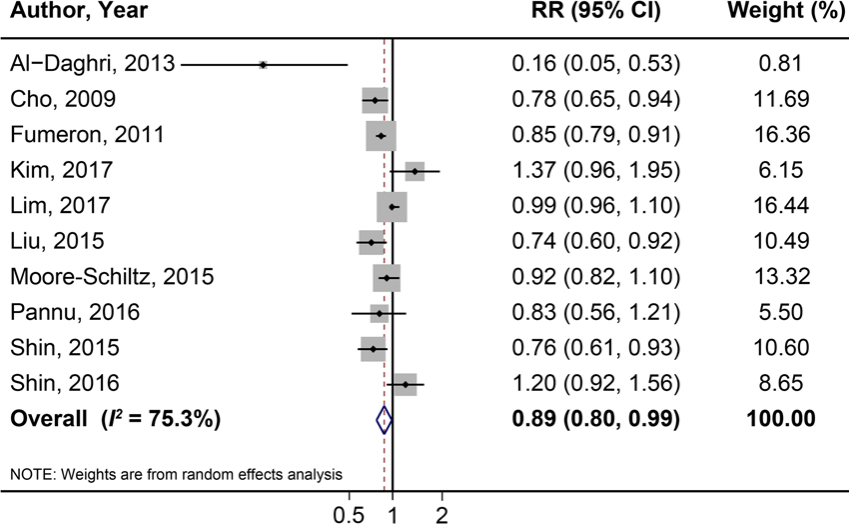
Forest plot of MetS for the highest *vs*. lowest category of dietary calcium intake.

The RRs (with 95% CI) of MetS for highest *vs*. lowest dietary calcium intake are shown in Fig. 2. Compared to lowest calcium intake levels, the overall RR for MetS among the highest levels was 0.89 (95% CI: 0.80–0.99), and the heterogeneity was estimated high (*I*^2^ = 75.3%). Neither Egger’s test (*P* = 0.46) nor the funnel plot (Supplementary Fig. 1) revealed probability of publication bias.

### Subgroup analyses

To identify the potential sources of heterogeneity, we performed subgroup analyses among all of the selected studies in order (Table 4). Notably, there is a significant association between dietary calcium intake and the risk of MetS only among female populations (RR: 0.74, 95% CI: 0.66–0.83), with no heterogeneity (*I*^2^ = 0). Moreover, association was more robust in the analysis based on cohort studies (RR: 0.82, 95% CI: 0.74–0.92; *I*^2^ = 31.3%) than that based on cross-sectional studies (RR: 0.92, 95% CI: 0.79–1.06; *I*^2^ = 73.7%). The associations were similar in other subgroups, including study location and the definition of MetS.

**Table 4:** Subgroup analyses of dietary intake of calcium and risk of MetS.

Subgroup	No.	RR (95% CI)	*I*^2^ (%)
**Design**			
Cohort	2	0.82 (0.74–0.92)	31.3
Cross-sectional	8	0.92 (0.79–1.06)	73.7
**Sex**			
Male	5	1.06 (0.82–1.37)	72.6
Female	5	0.74 (0.66–0.83)	0.0
**Location**			
Asia	6	0.92 (0.75–1.13)	80.6
US	2	0.84 (0.68–1.03)	63.1
**Definition of MetS**			
NCEP	8	0.91 (0.81–1.01)	72.2
IDF	3	0.69 (0.44 1.08)	73.0

### Dose-response analysis

**Figure 3:**
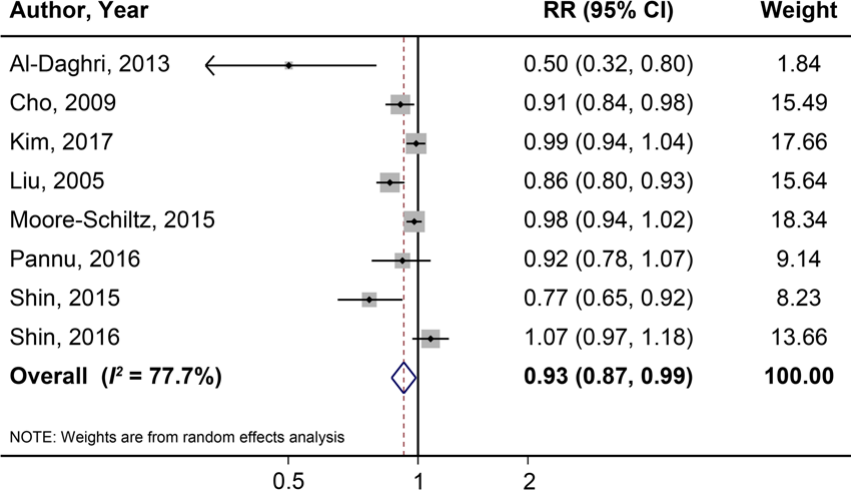
Dose-response meta-analysis of each 300 mg/day increment in dietary calcium intake and the risk of MetS.

For dose-response analysis, one cross-sectional study and one cohort study were excluded for lacking detailed data of dietary calcium intake level. We extracted data from the remaining 8 studies. The result showed that a 300 mg/day increase in dietary calcium intake was associated with a 7% lower risk of MetS (RR: 0.93; 95% CI: 0.87–0.99; *I*^2^ = 77.7%; see Fig. 3).

## Discussion

This systemic meta-analysis indicated that calcium intake may have a putative protective effect against MetS, which was also the most up-to-date epidemiological evidence. Assessment of dose-response effect showed a 300 mg/day increase in dietary calcium intake is significantly associated with a 7% decrease of MetS risk. In addition, subgroup analyses suggested the inverse association between dietary calcium intake and the risk of MetS was more robust in female population.

Metabolic syndrome is not a new medical condition. Eskil Kylin from Sweden reported the interesting observation of an aggregation of several metabolic risk factors^19^ in 1920s. However, the term “metabolic syndrome” was not formalized until 1998^20^. Other terms that are used synonymously with MetS such as syndrome X^21^, the deadly quartet^22^, and insulin resistance syndrome^23^. Among many lifestyle factors, dietary behavior is quite important in the development of MetS. Previous epidemiological studies suggested that particular kinds of dairy food including milk, yogurt, as well as total dairy food intake were inversely related to MetS or some of its components^24^. As we know, calcium is a major component of diary food. Although the underlying mechanisms remain incomplete, scientists postulated calcium makes a great difference in the beneficial effect against MetS^18,25^.

Similar to dairy food consumption, dietary calcium intake is inversely related to T2D, body weight, hypertension, glucose homeostasis, and cardiovascular disease^8,26–28^. In recent years, an increasing number of evidence - in particular, epidemiological studies - has suggested a potential link between calcium and MetS. Unfortunately, their results were inconsistent and controversial. To our knowledge, this review and meta-analysis is the first one designed to systematically explore the association between dietary calcium intake and MetS, including dose-response analysis.

In view of the current study, the controversy among different epidemiological studies may be attributed to gender difference. According to the data extracted from the Insulin Resistance Syndrome (DESIR) cohort, Drouillet *et al*. found that with increasing quartiles of dietary calcium intake, insulin concentrations and blood pressures decreased and HDL-cholesterol increased in women during about 9-year follow-up. In men, however, higher level of calcium intake was only significantly associated with 0.4 mmHg decrease in diastolic blood pressure^29^. Such difference was observed in several populations after being stratified by sex. Moore-Schiltz’s team suggested that men may require more calcium intake than current recommendation to gain the potential protective effect against MetS^13^ after analyzing the data from the National Health and Nutrition Exami nation (NHANES). In addition, Cho *et al*. indicated menopause status may influence the protective effect of calcium intake on MetS^10^. They found that only in postmenopausal women, higher level of calcium intake was related to decreased MetS risk. However, these findings weren’t repeated successfully in subsequent research^11^. Notably, heterogeneous methods to measure calcium intake, such as food frequency questionnaire (FFQ), 24-hour or 3-day food recall, were used among these studies, which may lead to misclassification of exposure and inconsistencies in findings.

Although the exact mechanisms remain unclear, the protective effect of calcium consumption on MetS may be due to its ability in regulating energy metabolism and insulin-stimulated glucose uptake and storage^30^. Accumulating evidence has suggested that imbalance of intercellular calcium homeostasis may play an important role in adipocyte function^6^. Increased cytosolic calcium promoted triglyceride accumulation as well as lipid storage through controlling de novo lipogenesis^31^. Additionally, unabsorbed calcium in the gastrointestinal tract isn’t without metabolic effects^32^. For example, calcium may form insoluble calcium soaps with fatty acids in gastrointestinal tract to influence fat and energy metabolism^33^.

This meta-analysis has several advantages. Above all, the primary advantage is that it is the first dose-response meta-analysis that quantitatively examined the relationship between dietary calcium intake and the risk of MetS. We also generated linear associations in addition to comparing the highest and lowest categories of calcium intake to increase the power of meta-analysis. Moreover, a common source of concern in meta-analysis is publication bias, but There was no evidence of publication bias in our study. Last but not least, relatively large number of study population enhanced the statistical power and made the subgroup analysis feasible.

On the other hand, the primary limitation of our analysis is that most of the selected studies were retrospective epidemiological studies. We also cannot exclude the possibility of confounding because of the observational nature of the included studies, although most of the original studies were adjusted for major potential confounders. Second, we also found significant heterogeneity in the result of the present study, and the sources of high heterogeneity cannot be totally explained by most of our subgroup analyses. However, significant differences in dietary calcium intake and MetS risk remained in several subgroups with lower heterogeneity, suggesting that the pooled results are likely reliable. Third, the number of included studies is relatively small. Besides, we might have neglected the missed unpublished reports, although every effort was made to estimate the unpublished risk. Finally, measurement and recall bias of dietary consumption assessment using food recall or FFQ would likely cause information bias.

In summary, this meta-analysis suggested that dietary calcium intake was inversely related to MetS risk, and such weak but significant association was supported by linear dose-response analysis. Subgroup analysis provided evidence that the beneficial effect may be significant among women, but less robust among men. In consideration of developing guidelines to prevent MetS by increasing the calcium-rich foods consumption, these findings may have important public health implications. However, the putative inverse association of dietary calcium intake with MetS risk still needs to be confirmed by larger prospective cohort studies and randomized clinical trials.

## Methods

### Search strategy

A comprehensive bibliographic research was performed on two databases - PubMed and Web of Science - for population-based studies published up to December 31th, 2018. Observational studies that examined the relationship between dietary intake of calcium and the risk of developing MetS were considered as potential suitable studies. The following terms were adopted as search strategy: calcium AND (“metabolic syndrome” OR “insulin resistance syndrome” OR “syndrome X”). To identify potential publications, we also examined the references of relevant reviews and meta-analysis. Bibliographic search was restricted to human studies without language limitation.

### Selection of articles

Each included studies in this meta-analysis should fulfill the following four criteria: (1) population-based epidemiological studies, including cohort studies, case-control studies, and cross-sectional studies; (2) the exposure of interest is intake of dietary calcium; (3) the outcome is MetS; (4) either adjusted risk ratios, including relative risks (RRs) and odds ratios (ORs) with 95% confidence intervals (95% CIs), or necessary data to calculate these values should be reported.

During the screening process, we excluded non-observational studies, studies that were not original (e.g., editorials, reviews, and commentaries), or not conducted in human subjects.

### Data extraction

Two investigators (authors DH and XF) extracted the relevant data independently from the identified articles using a standard extraction form. From each identified study, we extracted the following information: study characteristics (the study name, design, the publication year, name of first author, the country of data collection, and the number of follow-up years for cohort studies); the participants’ characteristics (sex, age, population size, and the incidence or prevalence of MetS); the method used to assess nutrient intakes; and the definition of metabolic syndrome measurements.

Quality assessment of included studies was conducted according to the Newcastle-Ottawa Scale (NOS) that gives a maximum of nine points for each study^34^, while cross-sectional studies were evaluated using a modified version of NOS with a highest score of ten^35^. Scores of 0–3, 4–6, and 7–9 were interpreted as low, moderate, or high quality, respectively.

### Statistical analysis

In the present study, we used RR with 95% CI to estimate risk; in addition, ORs (for case-control or cross-sectional studies) were used to directly determine the relative risk. Before inclusion in the overall analysis, we pooled the results that were stratified by sex by conducting a fixed effects meta-analysis.

We first pooled these RRs sequentially using the DerSimonian and Laird random effects method, taking both within-study and between-study variability into account^36^. Prospective and retrospective studies were analyzed separately in the main meta-analysis. To determine the sources causing between-study heterogeneity, we also performed subgroup analyses based on sex, location, study design, and the definition of MetS.

Dose-response analysis for calcium intake and MetS were conducted according to the method described by Greenland and Longnecker^37^ and using the publicly available Stata command written by Orsini *et al*.^38^. In accordance with this method, studies with three or more exposure categories were included. If the number of cases or participants was not available, we used variance-weighted least-squares regression to estimate risk^39^. If neither the median nor the mean was given in the original study, we used the categorical midpoint instead. If the lowest and/or highest category was open-ended, the midpoint of the category was determined by assuming that the width of the category is the same as the next adjacent category.

We used *I²* tests for across studies comparison. The values of 25%, 50%, and 75% were interpreted as low, moderate, and high degrees of heterogeneity, respectively^40^. Publication bias was evaluated by visually inspecting a contour-enhanced funnel plot and by performing Egger’s linear regression test (with significance defined as *P* < 0.10)^41^. Stata 12 (StataCorp LP, College Station, TX) was used to perform all statistical analyses. All statistical tests were two-sided and differences with a *P*-value < 0.05 were considered significant except where noted otherwise.

## Supplementary information

Supplementary data
