# Supplementary material for: Dietary Calcium Intake and the Risk of Metabolic Syndrome: A Systematic Review and Meta-Analysis
Source: Sci Rep. 2019 Dec 13;9:19046. doi: 10.1038/s41598-019-55507-x (PMC6911087; doi:10.1038/s41598-019-55507-x)

**Supplementary Data**

**Dietary Calcium Intake and the Risk of Metabolic Syndrome: A Systematic Review and Meta-Analysis**

Dan Han^1^, Xuexian Fang^2^, Danting Su^1^, Lichun Huang^1^, Mengjie He^1^, Dong Zhao^1^, Yan Zou^1^, Ronghua Zhang^1,*^

^1^Department of Nutrition and Food Safety, Zhejiang Provincial Center for Disease Control and Prevention, Hangzhou, China

^2^Department of Nutrition, School of Public Health, Zhejiang University School of Medicine, Hangzhou, China

*Corresponding author:

Ronghua Zhang, MD

Zhejiang Provincial Center for Disease Control and Prevention

Hangzhou, China

Email: rhzhang@cdc.zj.cn

**Supplementary Figure 1** Funnel plot of dietary calcium intake and the risk of MetS.


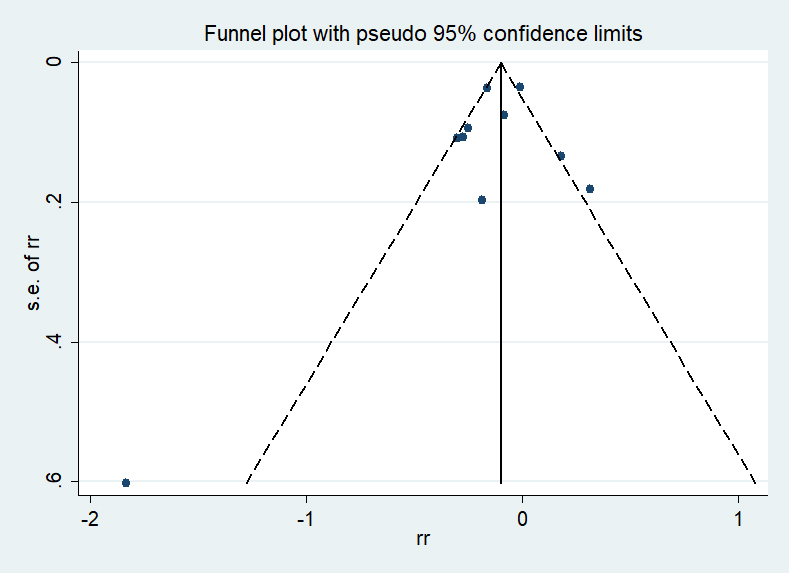

Supplement: Supplementary file 1 — Supplementary data [file 41598_2019_55507_MOESM1_ESM.docx]
